# NEIGHBORHOOD SEGREGATION, SOCIOECONOMIC STATUS, AND COGNITIVE FUNCTIONING AMONG OLDER CHINESE IMMIGRANTS

**DOI:** 10.1093/geroni/igad104.1409

**Published:** 2023-12-21

**Authors:** Fengyan Tang, Yanping Jiang, Ke Li, Andrea Rosso

**Affiliations:** University of Pittsburgh, Pittsburgh, Pennsylvania, United States; Rutgers University, New Brunswick, New Jersey, United States; University of New Hampshire, Durham, New Hampshire, United States; University of Pittsburgh, Pittsburgh, Pennsylvania, United States

## Abstract

The fast-growing population of older Chinese immigrants and their segregated residence highlight the importance of understanding the role of neighborhood context in cognitive health. The segregation-cognition association is equivocal based on a limited number of studies among Hispanic and Asian Americans. To close the knowledge gap, this study examined the associations of neighborhood segregation and socioeconomic status (NSES) with cognitive functioning among older Chinese immigrants. Four waves of cognitive performance tests were conducted in the Population Study of Chinese Elderly in Chicago (2011-2019) and linked to the 2010-2014 American Community Survey estimates of neighborhood contexts. NSES was a summary z-score of six census variables of education, income/wealth, and occupation. Neighborhood segregation was measured by the Index of Concentrations at the Extremes (ICE) that simultaneously assesses Chinese and English language use within a given census tract. There were 170 census tracts in the present sample of 2,044 participants. Latent growth curve models with adjusted cluster robust standard errors were estimated. On average, cognitive functioning declined over time (b= -0.07, p<.001). After adjusting for individual-level predictors, living in high-NSES neighborhoods was associated with slower cognitive decline (b=0.003, p=0.04). ICE was not associated with cognitive functioning, but boosted the protective effect of high NSES on cognitive decline (b=0.006, p=0.05). Neighborhood socioeconomic advantage was related to slower cognitive decline among older Chinese immigrants, especially among those living in neighborhoods with more English speakers or less segregation. This finding suggests complex associations between neighborhood context and cognitive health among Chinese immigrants.

